# Identification of Potential Biomarkers of Type 2 Diabetes Mellitus-Related Immune Infiltration Using Weighted Gene Coexpression Network Analysis

**DOI:** 10.1155/2022/9920744

**Published:** 2022-02-09

**Authors:** Ji Zhou, Xiaoyi Zhang, Lixia Ji, Guohui Jiang

**Affiliations:** Department of Pharmacology, School of Pharmacy, Qingdao University, Qingdao 266021, China

## Abstract

**Background:**

Type 2 diabetes mellitus (T2DM) is characterized by chronic low-grade inflammation, showing an increasing trend. The infiltration of immune cells into adipose tissue has been shown to be an important pathogenic cause of T2DM. The purpose of this study is to use the relevant database to identify some abnormally expressed or dysfunctional genes related to diabetes from the perspective of immune infiltration.

**Methods:**

Weighted gene coexpression network analysis (WGCNA) was employed to systematically identify the coexpressed gene modules and hub genes associated with T2DM development based on a microarray dataset (GSE23561) from the Gene Expression Omnibus (GEO) database. The key genes in modules highly related to clinical features were calculated and screened by using R software, and their participation in T2DM was determined by gene enrichment analysis. The mRNA levels of CSF1R, H2AFV, LCK, and TLR9 in pre-T2DM mice and normal wild-type mice were detected by WGCNA screening and real-time quantitative reverse transcriptase polymerase chain reaction (qRT-PCR).

**Results:**

We constructed 14 coexpressed gene modules, and the brown module was shown to be significantly related to T2DM. Through verification of the protein-protein interaction (PPI) network, four upregulated hub genes, CSF1R, H2AFV, LCK, and TLR9, were screened from the brown module and successfully distinguishedT2DM patients from healthy people. These hub genes may be used as biomarkers and important indicators for patient diagnosis. Enrichment analysis showed that these hub genes were highly associated with IL-6-related inflammatory metabolism, immune regulation, and immune cell infiltration. Finally, we verified the hub genes CSF1R, LCK, and TLR9 in a T2DM animal model and found that their mRNA levels were significantly higher in animals with T2DM than in control group mice (NC).

**Conclusions:**

In summary, our results suggest that these hub genes (CSF1R, LCK, and TLR9) can serve as biomarkers and immunotherapeutic targets for T2DM.

## 1. Introduction

Type 2 diabetes mellitus (T2DM) is an endocrine disease characterized by disorders of glucose, lipid, and protein metabolism [1]. According to the latest report from the Diabetes Atlas of the International Diabetes Federation (IDF), approximately 463 million diabetic patients (20-79 years old) existed worldwide in 2019, of which 116.4 million were in China, ranking first in the world [2]. It is estimated that by 2045, the number of people with diabetes will reach 702 million [3, 4]. T2DM has high morbidity and mortality rates as well as a high economic burden, and exploring the levels of genes associated with the occurrence and development of T2DM will provide important clues and strategies for early diagnosis and targeted therapies.

T2DM animals and patients have extremely active immune responses, especially the accumulation of many types of immune and inflammatory cells in visceral adipose tissue, which further reduces the body's sensitivity to insulin [5]. As classic immune cells, macrophages participate in inflammation in the body and in the progression of diabetes [6]. Studies have shown that pathogenic CD4^+^ and CD8^+^ T cells and CD11c^+^ M1 macrophages lead to the inflammatory infiltration and immune responses of macrophages, which in turn aggravate adipocyte apoptosis and peripheral insulin resistance [7, 8].

B and T cell infiltration is also essential for the inflammation of obesity-related adipose tissue [9, 10]. The infiltration of CD4^+^ immune cells eventually leads to the transformation of anti-inflammatory Tregs into proinflammatory Th1/Th17 cells, thus enhancing the proinflammatory function of macrophages and B cells in obesity-related T2DM [11–13]. At the same time, dendritic cells (DCs) stimulate the inflammatory circulation by regulating proinflammatory CD4^+^ T cells to initiate and regulate the recruitment of macrophages in adipose tissue [14]. In the adipose tissues of obese subjects, CD8^+^ T cells are activated to secrete the proinflammatory cytokine interferon-*γ*, and CD8^+^ T cells in turn promote the recruitment and activation of macrophages in this tissue [15]. Visceral adipose tissue is the key site of immune cell attack in subjects with T2DM, and a variety of immune cells cooperate with each other to activate the immune system and secrete a large number of proinflammatory cytokines, resulting in the body becoming insensitive to insulin [16]. Therefore, we propose that some key genes that affect the progression of T2DM are also abnormally expressed in immune cells.

With the rapid development of bioinformatics technologies, many tools have been developed to identify biomarkers [17–19]. Weighted correlation network analysis (WGCNA) is a systems biology method to describe the correlation patterns between genes across microarray samples, which is used to find highly related gene modules and calculate module membership to identify candidate biomarkers or therapeutic targets [20]. This algorithm has been widely used to identify biomarkers at the transcriptional level [21–23]. In this study, microarray data from public databases were subjected to WGCNA, and a coexpression network was constructed to screen differential genes between patients with T2DM and healthy people. Finally, the expression levels of the hub genes were verified in an animal model of T2DM. The results showed that the mRNA levels of CSF1R, LCK, and TLR9 in the pre-T2DM group were higher than those in the control group (NC).

## 2. Materials and Methods

### 2.1. Gene Data and Processing

One T2DM expression profile dataset was acquired from the Gene Expression Omnibus (GEO) database. Dataset GSE23561 was chosen for the selection of T2DM-related modules and genes and independently verified by animal experiments.

This dataset contained 17 groups of transcriptome data, which were used to construct a WGCNA coexpression network and to explore the differences in T2DM-related molecular mechanisms.

### 2.2. Construction of Weighted Gene Coexpression Networks

Using the above database to construct a gene coexpression network with R software, we identified key gene modules, explored the correlations between the gene modules and the disease phenotype, and then determined the hub genes in key modules. A coexpression network of 5000 genes was constructed with the WGCNA-R software package, and 7 was used as the soft threshold for the network. The weighted adjacency matrix was transformed into a topological overlap matrix (TOM) to estimate the network connectivity, and the hierarchical clustering method was used to construct the clustering tree from the TOM matrix. Then, all genes were classified according to the weighted coefficients, similar genes were divided into one module, and 5000 genes were successfully divided into 14 modules. The different colors represent the different modules, which are displayed on different branches of the cluster tree.

### 2.3. Identification of Key Modules and Hub Genes

First, the module-trait relationship diagram was constructed to represent the Pearson correlations between the gene modules and the disease phenotype. Then, the module most correlated with the T2DM phenotype was determined by the coefficient. Finally, the regions with a module membership (MM) > 0.8 and a gene significance (GS) score > 0.5 were screened to determine the hub genes in the key modules. We used an interactive gene search tool (STRING; https://string-db.org/) to construct the protein-protein interaction network [24] and Cytoscape (https://cytoscape.org/) to present the central node of the network node connectivity > 15 [25].

### 2.4. Functional Enrichment Analysis of Gene Modules

To determine the biological functions and signaling pathways associated with the occurrence and development of T2DM, annotation, visualization, and GO analyses were carried out using the Metascape database (https://www.metascape.org) [26]. A minimum overlap ≥ 3 and *p* ≤ 0.01 were considered to indicate statistical significance.

### 2.5. Relationships among Hub Genes, Immune Cell Infiltration, and Metabolism

The effects of genes on immune infiltration were evaluated. Single-sample gene set enrichment analysis (ssGSEA) was used to quantify the immune cell infiltration in each sample, and the Spearman correlation coefficient analysis revealed the correlations between genes and immune cells. At the same time, the effects of the genes on metabolism were evaluated. The correlations between gene expression and metabolic pathways were assessed by Kyoto Encyclopedia of Genes and Genomes (KEGG) pathway analysis.

### 2.6. Gene Set Variation Analysis

GSVA is a nonparametric and unsupervised method for evaluating transcriptome gene enrichment. By comprehensively scoring the genes of interest, the changes in the gene level are transformed into changes in the pathway level, and the biological functions of the gene is then determined. In this study, the gene set was downloaded from the molecular signature database (http://www.gsea-msigdb.org). The GSVA algorithm was used to score each gene set and thereby evaluate potential changes in the biological functions of different genes.

### 2.7. Establishment of the Pre-T2DM Model, Extraction of Total RNA, and qRT-PCR

Male wild-type C57BL/6J (6-weeks, 20-23 g) mice were purchased from Vital River Laboratory Animal Technology (Beijing, China) and housed at a constant temperature and humidity on a 12 h/12 h light/dark cycle. After one week of adaptation, the mice were randomly divided into two groups: the normal group (*n* = 10) and the pre-T2DM group (*n* = 10). The mice in the normal group were fed a routine diet, while the mice in the pre-T2DM group were fed a 60% high-fat diet for 12 weeks to induce visceral adipose tissue obesity, insulin resistance, and high blood glucose levels.

After anesthesia, the epididymal adipose tissues of the mice were removed and quickly preserved in liquid nitrogen. Then, total RNA was extracted from the epididymal adipose tissues with the TRIzol reagent (CWBIO, Beijing, China) and reverse transcribed into cDNA with a reverse transcription kit (Tiangen, Beijing, China). The TB Green Premix Ex Taq II kit (TaKaRa, Dalian, China) was used for qPCR. The selected primers were designed by TSINGKE (Qingdao, China), and their sequences are shown in Table 1.

### 2.8. Statistical Analysis

Statistical analysis was carried out with R software (version 3.6). The bilateral statistical test was used, and *p* < 0.05 indicated statistical significance [27]. All results are expressed as the mean ± standard deviation. Animal experiment statistical analyses were performed using Student's *t*-test with Prism 8 software.

## 3. Results

### 3.1. Dendrogram and Trait Heatmap

**Figure 1:**
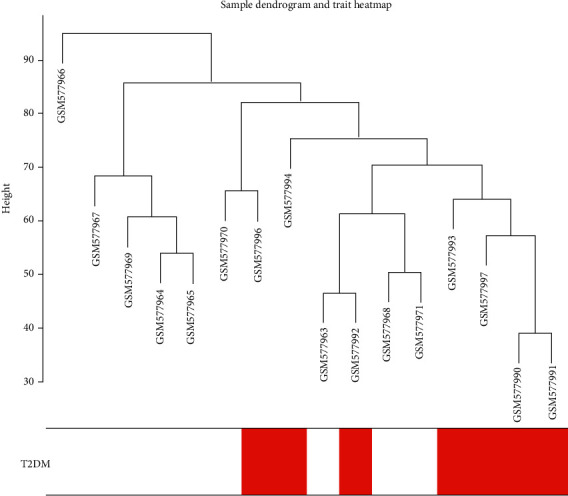
Clustering tree and heatmap of the intercepted samples.

The GSE23561 matrix data were downloaded from the public Gene Expression Omnibus (GEO) database. The transcriptional data of the 17 groups included the healthy control group (*n* = 9) and the T2DM group (*n* = 8). These data were used to construct a WGCNA coexpression network and to explore the differences in T2DM-related molecular mechanisms. The outlier samples were deleted according to the results of the clustering tree (Figure 1).

### 3.2. Gene Coexpression Networks of T2DM

**Figure 2:**
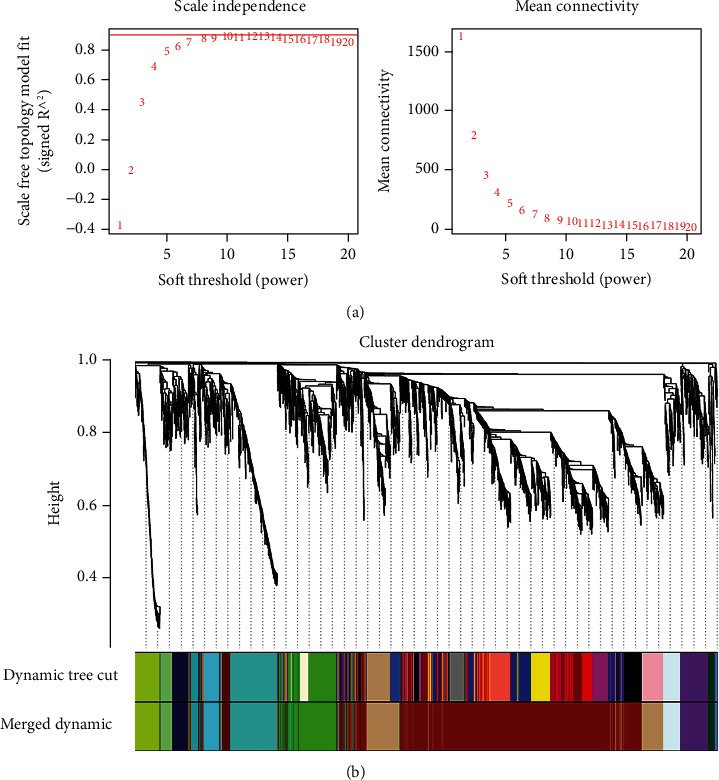
Construction of the gene coexpression network: (a) analysis of the soft threshold power (*β*) from 1 to 20; (b) all genes are divided into 14 modules with different colors.

The top 5000 genes with the largest variance were selected by the WGCNA-R software package and used to construct a coexpression network for analysis. To build a scale-free network, one of the key factors in creating a WGCNA is the soft threshold power. We selected *β* = 7 as the soft threshold power (Figure 2(a)). We completed the network topology analysis with a threshold power ranging from 1 to 20. When the power value was equal to 7 (scale-free *R*^2^ = 0.9) [21], our record showed a scale-free gene coexpression network with complete module characteristics. The cluster tree structure of the topology overlap matrix was constructed. The different branches and colors of the cluster tree represent the different gene modules. The coexpression network of 5000 genes established by WGCNA was divided into 14 modules for follow-up analysis (Figure 2(b)). Genes in the 14 coexpression modules ranging from 2 to 2316 are shown in Table 2.

### 3.3. Identification of Key Modules and Enrichment Analysis

**Figure 3:**
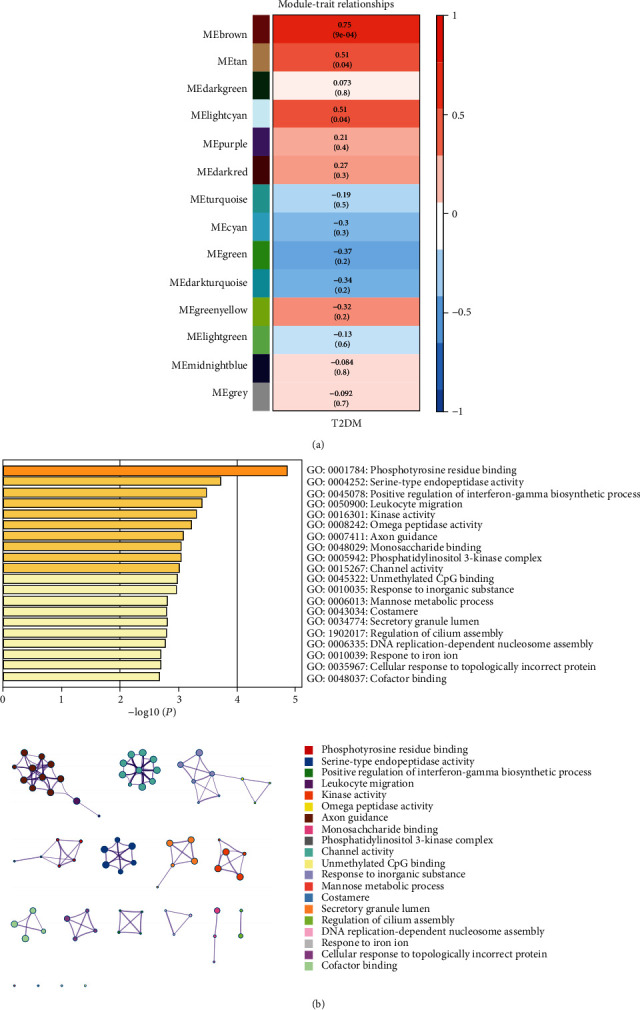
Key modules and feature notes. (a) Heatmap showing that the brown module was associated with clinical features. (b) The top 20 enriched functions in the brown module are shown in the bar chart. The network diagram was constructed with each enrichment item as the node and the node similarity as the edge.

Among the 14 modules, we calculated the correlation coefficient between each module and the phenotype to determine the module most relevant to clinical characteristics. Among them, both the brown module (*R*^2^ = 0.75, *p* = 9*e* − 04) and the light blue module were highly correlated with T2DM (*R*^2^ = 0.51, *p* = 0.04) (Figure 3(a)). The correlation coefficients between the other modules and T2DM were less than 0.5. Finally, we chose the brown module, which was most correlated with T2DM, as the key module. Then, the genes contained in the module were analyzed by using Metascape to elucidate the associated biochemical pathways involved. The 20 most abundant terms were related to immune activation and carbohydrate metabolism (Figure 3(b)).

### 3.4. Identification and Validation of Hub Genes and Network Analysis

**Figure 4:**
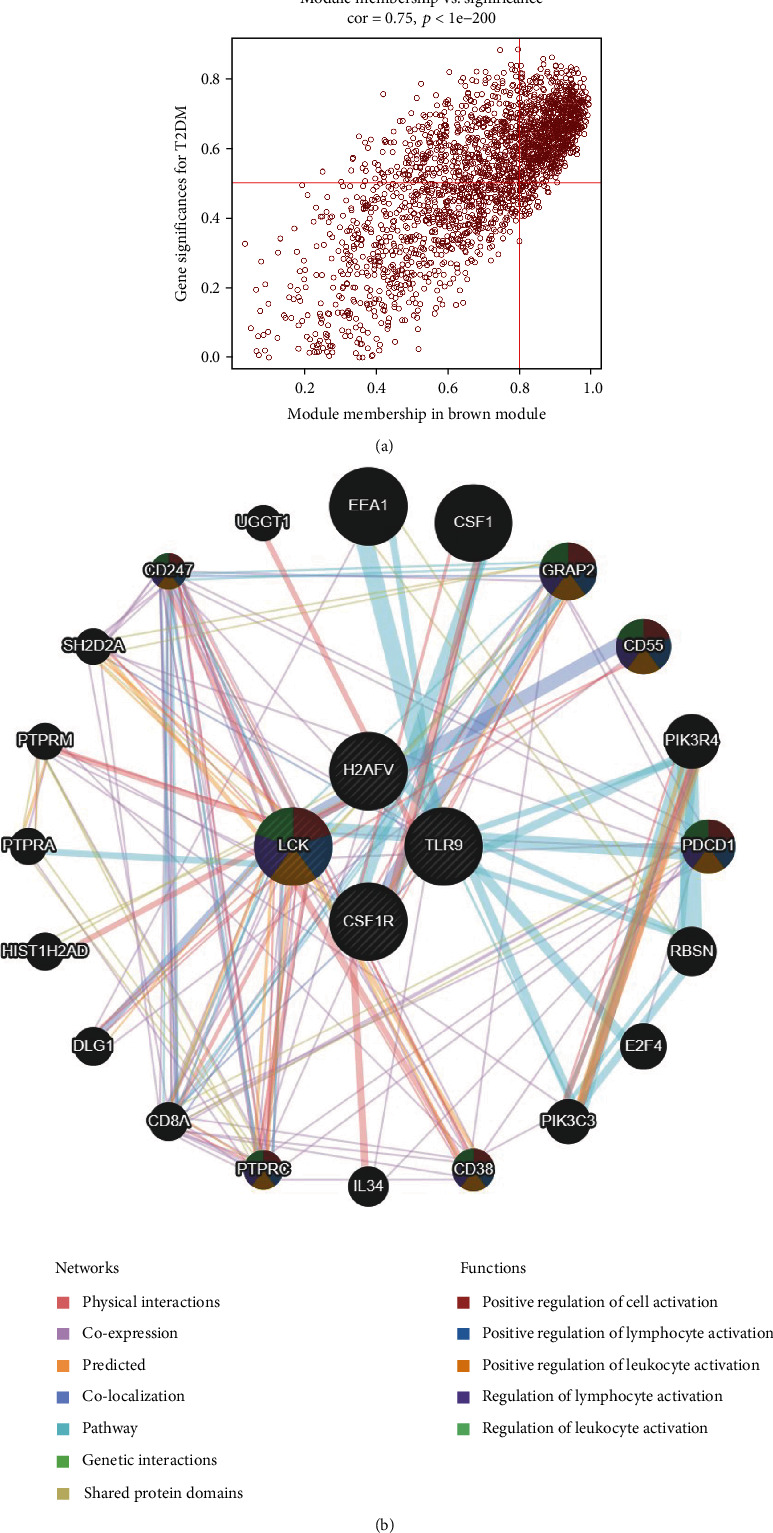
Identification of hub genes. (a) Scatter plot of brown module genes. Each brown dot represents a gene, and the dots in the red box represent the genes with a module membership > 0.8 and a gene significance score > 0.5. The genes in the red region group are shown in the upper right corner. (b) GeneMANIA network construction. As shown in the figure, the different functions of each gene are represented by different colors in each node.

Because the brown module was the most correlated with the diabetes phenotype and the most likely to participate in the occurrence and development of diabetes, it was selected for further study. In addition, 890 key genes in the brown module were screened according to their module membership and gene significance values (Figure 4(a)). Furthermore, in the PPI network, a connectivity > 15 was utilized to identify multiple genes as central nodes, and Cytoscape was used to visualize these results (Figure S1). Finally, four genes (LCK, H2AFV, TLR9, and CSF1R) were selected as hub genes. Furthermore, the relationships between these hub genes and T2DM were assessed to reveal their potential roles in T2DM. Then, to determine the importance of the hub genes and analyze the network in the corresponding module, we used GeneMANIA (http://genemania.org/) to further verify and analyze the identified hub genes. The network formed by the brown module contained 24 key genes, including 4 central dynamic genes and 20 peripheral predicted genes. A frequent and extensive interaction network was constructed according to the Pearson correlation scores (Figure 4(b)). The relationships between these hub genes and T2DM were further revealed to show the potential roles of these key genes in T2DM.

### 3.5. Relationships of the Hub Gene Levels with Those of Immune Cell Infiltration and Metabolism

**Figure 5:**
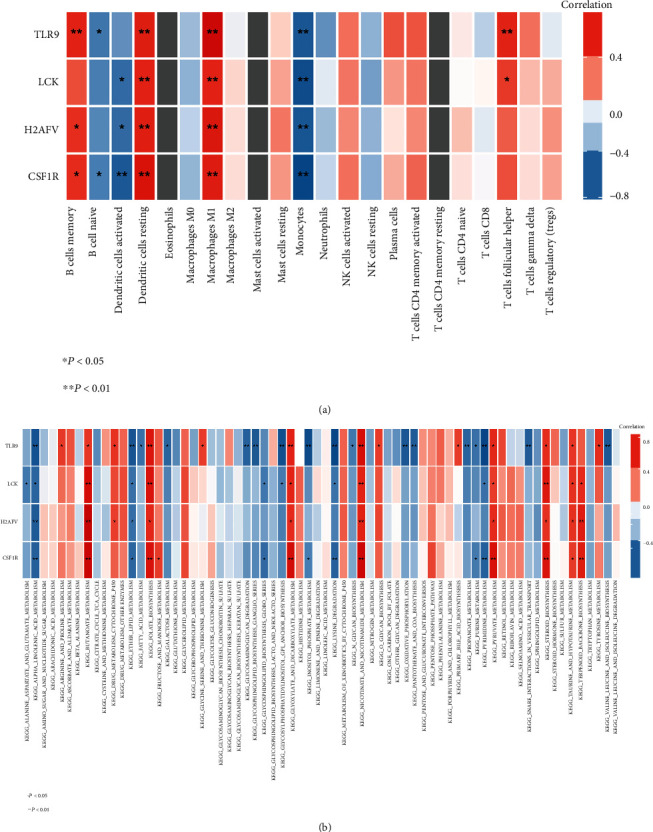
Correlations between hub genes and the profiles of immune infiltration and metabolic pathways. (a) Correlation map of the hub genes with 20 types of immune cells. Positive correlations are shown in red, and negative correlations are shown in blue. Shadow colors and asterisks represent the values of the corresponding correlation coefficients. ^∗^*p* < 0.05, ^∗∗^*p* < 0.01. (b) The correlation map between hub genes and multiple metabolic pathways. Positive correlations are shown in red, and negative correlations are shown in blue. The rectangular color and asterisk represent the value of the corresponding correlation coefficient. ^∗^*p* < 0.05, ^∗∗^*p* < 0.01.

The correlation between key genes and the level of immune infiltration in each patient was analyzed to explore the relationship between key genes and the level of immune infiltration. The results showed that all four hub genes were significantly correlated with the degree of immune cell infiltration (Figures 5(a)). For example, the expression of LCK was positively correlated with the levels of M1-like macrophage and memory B cell infiltration. The expression of TLR9 was negatively correlated with the infiltration of activated monocytes and dendritic cells. Similar results were found for the other hub genes. Thus, LCK, TLR9, CSF1R, and H2AFV are suggested to play important roles in immune infiltration in subjects with diabetes.

Because abnormal metabolism also plays an important role in the progression of diabetes, we studied the expression levels of key genes associated with metabolism in diabetes and identified four hub genes closely related to the metabolic process (Figure 5(b)). For example, the expression of CSF1R was positively correlated with the levels of butanoate metabolism and folate biosynthesis, which can affect the metabolism of intestinal microorganisms and induce immune stress [28–31]. Similar results were found for the other three hub genes. Thus, LCK, TLR9, CSF1R, and H2AFV are suggested to play an important role in the metabolism of patients with diabetes.

### 3.6. Molecular Characterization of Hub Genes

**Figure 6:**
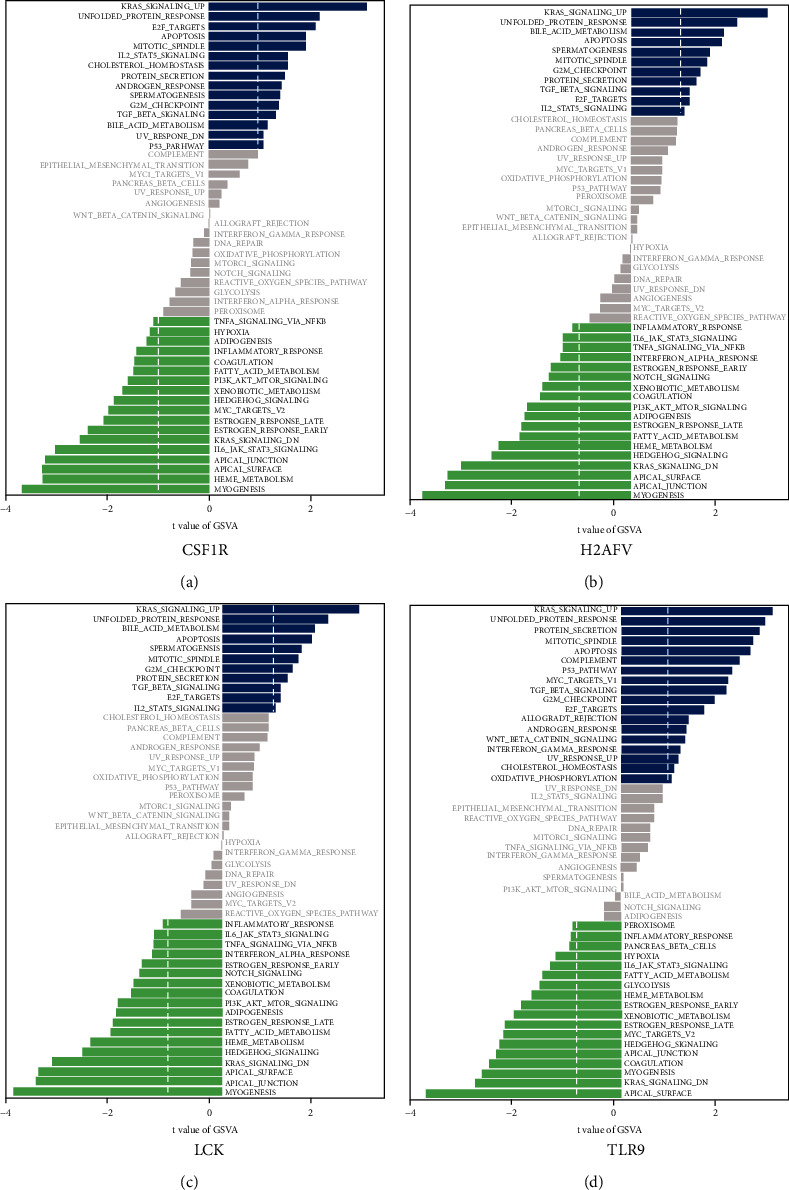
GSVA of the hub genes: (a) GSVA bar chart of CSF1R; (b) GSVA bar chart of H2AFV; (c) GSVA bar chart of LCK; (d) GSVA bar chart of TLR9.

To further explore the potential functions of the hub genes in diabetes, we used their comprehensive scores for GSVA analysis, and the samples were divided into CSF1R/H2AFV/LCK/TLR9^LOW^and CSF1R/H2AFV/LCK/TLR9^high^ groups. In CSF1R samples with relatively high expression, multiple pathways were enriched, such as myogenesis, IL6-JAK-STAT3 signaling, PI3K-AKT signaling, and inflammatory response, which were significantly associated with diabetic inflammation (Figure 6(a)). Similarly, H2AFV was associated with adipogenesis and TNF-A signaling (Figure 6(b)), but negative correlations were observed between mitotic spindles and other pathways in the samples with relatively low LCK expression (Figure 6(c)). Similarly, inflammation-related pathways and glycolysis were enriched in the samples with relatively high TLR9 expression (Figure 6(d)). GSVA showed that CSF1R/H2AFV/LCK/TLR9 is associated with various pathways related to diabetes.

### 3.7. Validation of the Hub Genes in Mice

**Figure 7:**
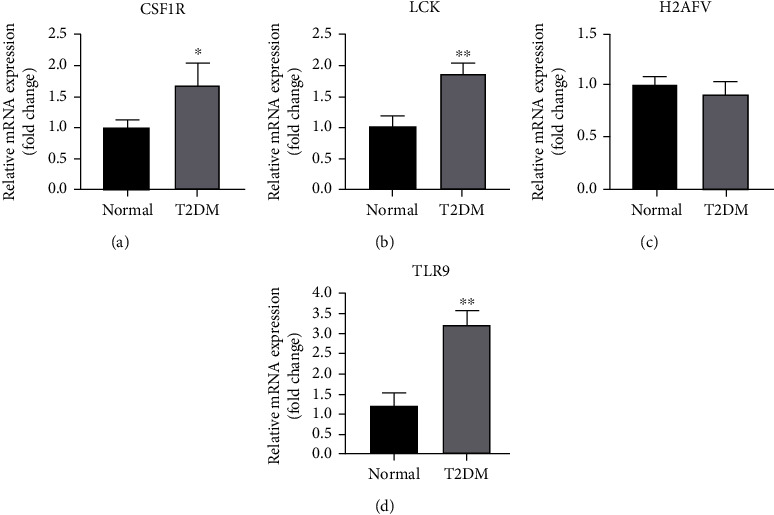
Verification of the hub genes. Total RNA was extracted from the epididymal adipose tissues of obese mice and wild-type control mice for qRT-PCR analysis. The expression of CSF1R, H2AFV, LCK, and TLR9 in the mice (*n* = 3 per group). ^∗^*p* < 0.05, ^∗∗^*p* < 0.01, and ^∗∗∗^*p* < 0.001 versus the control. The data are presented as the mean ± SEM.

We further proved the reliability of the results by detecting the expression levels of the hub genes in the epididymal adipose tissues of obese mice by qRT-PCR. The results showed that the expression levels of CSF1R, LCK, and TLR9 of the mice fed HFD for 12 weeks were increased compared with those of normal mice. Generally, the qRT-PCR results obtained for the obese mice were consistent with those of the analysis (Figure 7).

## 4. Discussion

Changes in immune cells induce a stress response, and T2DM is a disease involving both immune and metabolic pathways. Chronic low-grade inflammation of various organs in the body can mediate the development of type 2 diabetes and further cause related complications and endanger life [32, 33]. The factors affecting the pathogenesis of T2DM have been widely discussed, revealing that the genes associated with immune cell infiltration and carbohydrate metabolism affect the degree of inflammation and the dynamic balance of islet function, thereby playing an important role in the onset and development of T2DM [5]. However, we herein used WGCNA to verify the effects of key genes on immune cell infiltration in T2DM, and the functions of immune cells were shown to be altered in patients with T2DM [34]. Therefore, these hub genes may have important clinical significance and serve as biomarkers or therapeutic targets for research and prediction. Here, the GSE23561 dataset, including a normal group (*n* = 9) and a T2DM group (*n* = 8), was selected for bioinformatics analysis. This study provides an effective and reliable coexpression network that can potentially be used for T2DM research.

In this study, we analyzed the relationships between the normal group and T2MD group via a module constructed by a coexpression network. Then, four hub genes related to the occurrence and development of diabetes were selected from the brown module: CSF1R, H2AFV, LCK, and TLR9. At the same time, the patients were divided into an upregulated T2DM group and a downregulated normal group, and the functions of key genes were predicted by GSVA. Thus, this study determined the effects of immune cell infiltration and potential genes related to disease progression on patients and provided a new perspective for studying dysfunction in patients with diabetes from the perspective of immunity.

Among the four hub genes we screened, CSF1R is a receptor for the colony-stimulating factor CSF1 and has been shown to play an important role in many chronic metabolic diseases [35–39]. In our study, CSF1R was overexpressed in T2DM, and GSVA showed that CSF1R was involved in myogenesis, inflammatory signaling pathways, and adipogenesis. Studies have confirmed that the selective ablation of NK cells expressing CSF1R can prevent obesity and insulin resistance [35]. CSF1R signaling can also regulate the proliferation, differentiation, and function of macrophages such as microglia and participate in the regulation of microglial homeostasis and inflammation [36]. The pharmacological inhibition of CSF1R is beneficial for subjects with Alzheimer's disease [37]. CSF1R also plays an important role in the innate immune response, and CSFIR/CSF1 plays an important nutritional role in kidney and muscle growth and repair [39]. At the same time, our study results suggest that the expression of CSF1R is related to different degrees of immune cell infiltration. The results revealed a significant relationship between the expression of CSF1R with both M1 macrophages and memory B cells.

LCK is a member of the Src family of protein tyrosine kinases (PTKs) [40]. It is the first kinase activated downstream of the TCR signaling pathway and a key factor in initiating the TCR signaling pathway. It plays a key role in the activation, development, and proliferation of T cells [41–43]. LCK is mainly expressed in T cells and NK cells, and it has a special sequence that regulates the activity of kinases [44]. In this study, we observed the enrichment effect of LCK on key pathways related to diabetes, such as the PI3K signaling pathway and the inflammatory response pathway. Singh et al. found that LCK can bind to the C-terminus of CD4 or CD8 through N-terminal cystine to form an LCK-CD4/CD8 complex, which induces the antigen-presenting cell-mediated activation of CD4^+^ and CD8^+^ T cells [45]. In human T lymphocytes, the activation of LCK components can promote the secretion of IL-2 and activate the PI3K signaling pathway, causing a series of inflammatory responses, which is consistent with our results. In 2015, Gurzov et al. reviewed the interactions between protein tyrosine phosphatase and Src kinases, such as LCK, and determined their roles in autoimmune-mediated diabetes [46]. In the same year, Patry et al. reported that the inhibition of LCK inhibited T cell activation in patients with type 1 diabetes, supporting the use of LCK inhibition in the treatment of type 1 diabetes [47]. We observed that the abovementioned experimental results were consistent with our genetic analysis, which provides a good strategy for exploring the relationship between the role of LCK in immunity and diabetes. Finally, we speculate that the high expression of LCK may lead to changes in the immune system, lead to proinflammatory M1 macrophage infiltration, promote the development of inflammation, and eventually lead to chronic metabolic diseases.

Toll-like receptor 9 (TLR9), a member of the TLR family, which recognizes single-stranded DNA containing unmethylated CpG motifs, is mainly distributed on the endoplasmic reticulum (ER) membranes of different immune cells, such as macrophages, dendritic cells, B cells, and T cells [48, 49]. TLR9 is also highly expressed in plasma cell-like dendritic cells (pDCs), which is consistent with our results. TLR9 is closely related to the expression of B cells and DCs, and the expression of TLR9 is related to the degree of immune infiltration. Nishimoto et al. reported that the release of DNA (CfDNA) from free cells is caused by obesity-related adipocyte degeneration, which leads to the accumulation of macrophages in adipose tissue through TLR9 [50]. Ghosh et al. demonstrated that, compared with WT mice, TLR9-/- mice fed a HFD easily gained weight and exhibited decreased glucose tolerance and insulin resistance [51]. At the same time, GSVA of TLR9 revealed that the high expression group was mainly associated with the regulation of peroxisomes, inflammatory stress, glycolysis, and islet *β* cells. TLR9 may lead to impaired glucose tolerance and islet dysfunction by affecting the glucose uptake in diabetic adipose tissue and the malignant induction of adipose tissue. It was also shown to promote the infiltration of immune cell groups of memory B cells and dendritic cells.

By assessing immune cell infiltration in diabetes, we identified four positively related immune cell types: memory B cells, M1 macrophages, T cell follicle helper cells, and resting dendritic cells. In most T2DM patients, macrophages are reportedly activated by LPS and differentiate into proinflammatory M1-like cell phenotypes, which stimulate the inflammatory response, leading to the development of chronic inflammation [52]. Dendritic cells are reported to play a critical role in initiating and maintaining the immune response in subjects with T1DM [53]. The other three negatively correlated immune cells were natural B cells, activated dendritic cells, and monocytes. This result paves the way for further exploring diabetes in the context of immunity, which is worthy of in-depth exploration.

Finally, we analyzed the possible biological processes by GSVA and found some activated pathways that were highly correlated with immune activation, inflammation, glucose and lipid metabolism, islet *β* cell disorder, interleukin-6 production, fatty acid metabolism, and glycolysis. Therefore, our results confirm that the infiltration of immune cells and the development of inflammation may play key roles in the progression of T2DM. We also found that some pathways related to the cell cycle and mitosis were inhibited, which may indicate that patients with high expression are at a higher risk for growth inhibition.

## 5. Conclusions

In summary, we used a publicly available GEO dataset to perform WGCNA of immune cell infiltration in the context of diabetes and systematically identified a module related to clinical features and four hub genes (CSF1R, H2AFV, LCK, and TLR9) that may be associated with T2DM. Immune infiltration and qPCR verification analyses showed that three of these genes (CSF1R, LCK, and TLR9) may affect immune cell infiltration and nutritional metabolism through a variety of biological functions and pathways, thus affecting the progression of T2DM. Our findings will contribute to further understanding the pathogenesis of T2DM and may provide new insights into its immune pathogenesis.

## Figures and Tables

**Table 1:** PCR primers for quantitative real-time PCR.

Gene	Primer sequence (5′ → 3′)	
CSF1R	CCTGAAGGTGGCTGTGAAGATG	GCTCCCAGAAGGTTGACGATG
H2AFV	GGAGTCAGATTAAAGGA	TCAAGGCATCAGGTAAGG
LCK	CACGGATGACAGCTCTGAAA	ATGGAGAACGGGAGCCTAGT
TLR9	ATGGACGGGAACTGCTACTAC	CATTGGTGTGGGTGATGCTTT
GAPDH	GATACTGCACAGACCCCTCCA	GCAGTTCCGGTCATTGAGGTA

**Table 2:** Modules and number of genes in each module.

Brown 2316	Cyan 156	Dark green 62	Dark red 82	Dark turquoise 55
Green 438	Greenish yellow 230	Gray 2	Light cyan 153	Light green 114
Midnight blue 154	Purple 242	Tan 563	Turquoise 433	

## Data Availability

We wish not to share the raw data as the authors are aiming for future publications from the data. However, the data used to support the findings of this study are available from the corresponding author upon request.

## Supplementary Materials

Supplementary Materials Figure S1: protein-protein interaction (PPI) gene network in the brown module. The size and color of the circle represent the number of genes enriched, and the genes with dark color were selected for analysis. The network was built with Cytoscape software.
