# Supplementary material for: Identification of Potential Biomarkers of Type 2 Diabetes Mellitus-Related Immune Infiltration Using Weighted Gene Coexpression Network Analysis
Source: Biomed Res Int. 2022 Feb 9;2022:9920744. doi: 10.1155/2022/9920744 (PMC8849810; doi:10.1155/2022/9920744)
Supplement: Supplementary Materials — Figure S1: protein-protein interaction (PPI) gene network in the brown module. The size and color of the circle represent the number of genes enriched, and the genes with dark color were selected for analysis. The network was built with Cytoscape software. [file 9920744.f1.zip › Figure S1.pdf]

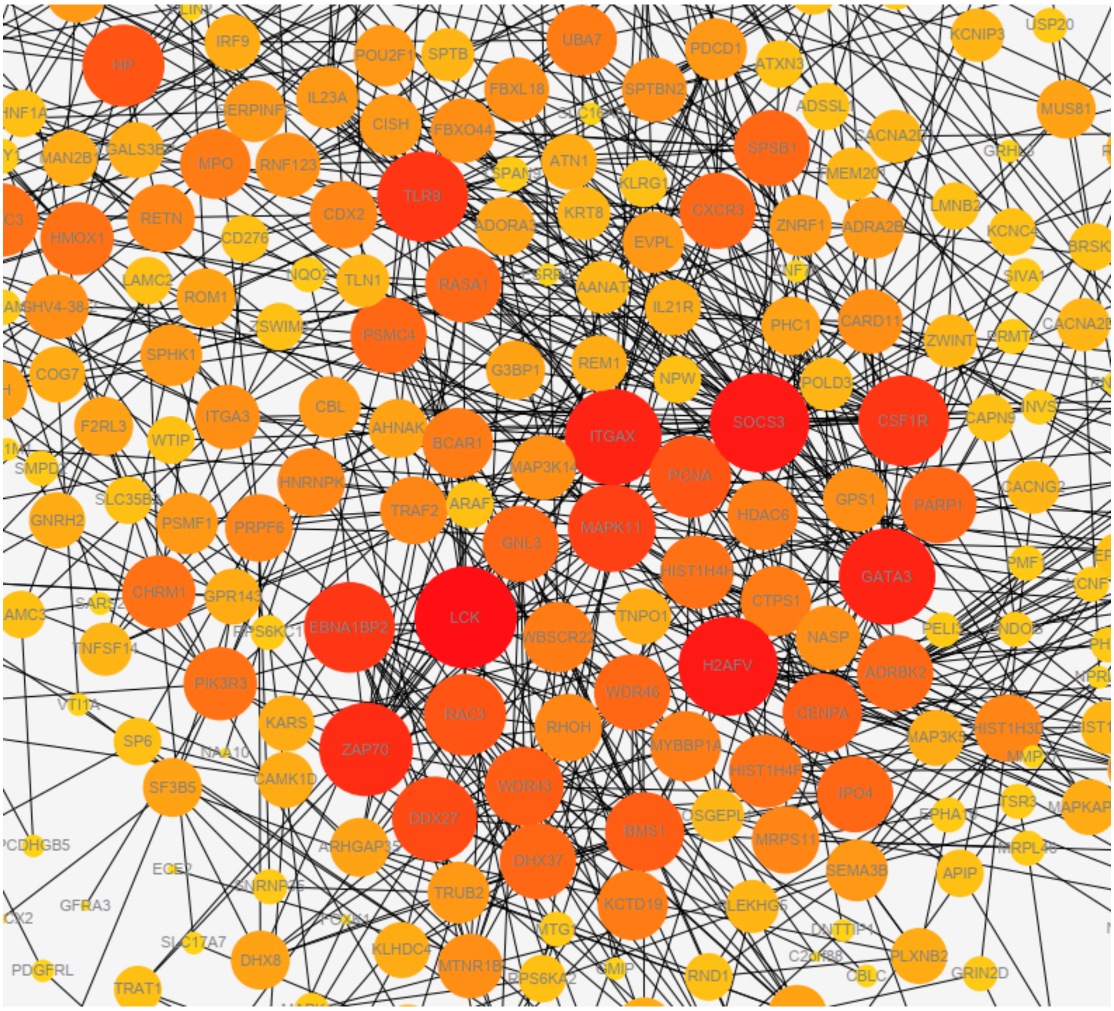

Figure S1. Protein–protein interaction (PPI) gene network in the brown module.The size and color of the circle represent the number of genes enriched, and the genes with dark color were selected for analysis. The network was built with Cytoscape software.
